# Pro-Inflammatory and Anti-Inflammatory Interleukins in Infectious Diseases: A Comprehensive Review

**DOI:** 10.3390/tropicalmed9010013

**Published:** 2024-01-04

**Authors:** Arwa A. Al-Qahtani, Fatimah S. Alhamlan, Ahmed Ali Al-Qahtani

**Affiliations:** 1 Department of Family Medicine, College of Medicine, Al-Imam Mohammad Ibn Saud Islamic University, Riyadh 11432, Saudi Arabia; arahalqahtani@imamu.edu.sa; 2 Department of Infection and Immunity, King Faisal Specialist Hospital & Research Center, Riyadh 11211, Saudi Arabia; falhamlan@kfshrc.edu.sa; 3 Department of Microbiology and Immunology, College of Medicine, Alfaisal University, Riyadh 11211, Saudi Arabia

**Keywords:** interleukins, infectious diseases, pro-inflammatory, anti-inflammatory, immune response, diagnosis, therapy

## Abstract

Interleukins (ILs) are signaling molecules that are crucial in regulating immune responses during infectious diseases. Pro-inflammatory ILs contribute to the activation and recruitment of immune cells, whereas anti-inflammatory ILs help to suppress excessive inflammation and promote tissue repair. Here, we provide a comprehensive overview of the role of pro-inflammatory and anti-inflammatory ILs in infectious diseases, with a focus on the mechanisms underlying their effects, their diagnostic and therapeutic potential, and emerging trends in IL-based therapies.

## 1. Introduction

Infectious diseases remain a major public health concern, causing significant morbidity, and continue to be a significant public health issue globally [1]. These diseases are caused by disease-causing microorganisms like viruses, bacteria, fungi, and parasites, which can enter the body through various means, such as ingestion, inhalation, or contact with contaminated surfaces or bodily fluids. Entry of infectious pathogens into the host causes the immune system to activate a complex defense mechanism involving the activation of various immune cells as well as the production of different signaling molecules, including cytokines. Interleukins are a type of cytokine that plays a crucial role in modulating the immune response to infectious agents. Interleukins are produced by different immune cells such as B cells, T cells, dendritic cells, and macrophages. These immune molecules act on different target cells, including immune cells, endothelial cells, and epithelial cells. Several interleukins are involved in the immune response to infectious agents, and these can be classified as either pro-inflammatory or anti-inflammatory depending on their effects on the immune system.

Pro-inflammatory interleukins, such as interleukin-1β (IL-1β) and IL-6, are essential for initiating the immune response to infectious agents. They stimulate immune cell recruitment and activation, increase their vascular permeability, and induce fever, all of which are critical for fighting off invading pathogens. However, excessive secretion of pro-inflammatory interleukins can lead to a hyper-inflammatory response, which can cause tissue damage and contribute to the development of severe infections and sepsis [2,3,4].

In contrast, anti-inflammatory interleukins, such as IL-10 and transforming growth factor-beta (TGF-β), play an important role in limiting inflammation and preventing tissue damage [5,6]. They inhibit the production of pro-inflammatory cytokines and promote differentiation and activation of regulatory immune cells, which can help to control the immune response and promote tissue repair [7,8]. However, an excessive anti-inflammatory response can also be detrimental, as it can lead to immune suppression and impaired pathogen clearance.

Interleukins can significantly influence the immune response and vaccine efficacy. For instance, interleukins like IL-2 or IL-15 are used to stimulate the immune system, enhancing the activation and proliferation of immune cells when incorporated into vaccine formulations [9]. In addition, interleukins often serve as adjuvants, substances added to vaccines to boost their effectiveness. For example, IL-33 has been found to improve CD8+ T cell response during chronic infection while its incorporation as a vaccine adjuvant was found to enhance humoral response in a DNA-based human immunodeficiency virus (HIV) vaccine [10,11]. Thus, by improving the body’s immune memory and promoting a stronger and longer-long-lasting response, interleukins may contribute to more potent vaccines. Furthermore, interleukins are critical in shaping the balance between Th1 and Th2 immune responses, which is crucial for generating the appropriate type of immunity needed for a particular vaccine [12]. Vaccines targeting intracellular pathogens may require a Th1-biased response, while those against extracellular pathogens may necessitate a Th2-biased response. Here, interleukins help fine-tune this balance to optimize vaccine outcomes.

Interleukins also influence the development of memory T cells, which are essential for long-term immunity. This underscores the pivotal role that interleukins play in shaping the adaptive immune response. Memory T cells are a specialized subset of T lymphocytes that recognize previous encounters with pathogens, allowing the immune system to respond more rapidly and effectively upon re-exposure. Interleukins, such as IL-7 and IL-15, are critical in promoting the formation and maintenance of memory T cells by supporting the survival, proliferation, and differentiation of these cells [13,14]. Memory T cells are crucial for sustaining immunity over time due to their protection against recurring infections, including viral infections like influenza and bacterial infections like tetanus [15,16]. Understanding the influence of interleukins on immune cell development is not only fundamental to our comprehension of immune response but also holds significance in drug design for infectious diseases to ensure sustained protection against infectious diseases.

The balance between the levels of pro-inflammatory and anti-inflammatory interleukins is crucial for the effective management of infectious diseases. An imbalance in the production of these interleukins can also lead to the development of various infectious and autoimmune diseases. Therefore, understanding the regulation of interleukins in infectious diseases is crucial for the development of new therapeutic strategies that target these molecules.

Here, we highlight the roles played by interleukins in infectious diseases, with a focus on their pro-inflammatory and anti-inflammatory effects. We will discuss the various interleukins involved in the immune response to infectious agents, their mechanisms of action, and their potential therapeutic implications. This review will help to enhance our understanding of the complex interaction between the immune system and infectious agents. We also highlight the possibilities of new therapeutic strategies to combat infectious diseases.

## 2. Pro-Inflammatory Interleukins

Pro-inflammatory interleukins are cytokines that are produced in response to infectious agents and other inflammatory stimuli (Table 1). This section will focus on two main families of pro-inflammatory interleukins: the IL-1 family and the IL-6 family.

### 2.1. IL-1 Family

The IL-1 family of cytokines is made up of 11 members, including IL-1α, IL-1β, IL-18, IL-33, IL-36α, IL-36β, IL-36γ, IL-36Ra, IL-37, IL-38, and the IL-1 receptor antagonist (IL-1Ra). These cytokines are secreted by different cell types, including macrophages, monocytes, dendritic cells, and epithelial cells, and they exert their effects by binding to specific receptors [1,2,3]. IL-1 family cytokines initiate signaling cascades that activate immune cells and stimulate the secretion of other pro-inflammatory cytokines, amplifying immune response [4,5,6].

IL-1 family cytokines exert their effects through binding appropriate receptors, initiating intracellular signaling pathways. This triggers the activation of transcription factors, leading to inflammatory gene expression, immune cell recruitment, and tissue repair [7,8,9]. IL-1 family cytokines also regulate cell survival, proliferation, and differentiation, thereby modulating immune responses to infectious agents [1,7,10].

#### 2.1.1. Role of IL-1 Protein Family in Immune System Activation during Infection

IL-1β, a prominent member of the IL-1 family, is involved in initiating and amplifying the immune response to infectious agents. It is involved in immune cell recruitment and activation, production of pro-inflammatory cytokines, and modulation of adaptive immunity [14,15,16]. IL-18 contributes to the immune response against viral infections by activating T cells and natural killer (NK) cells, promoting their cytotoxic activity [17]. IL-1 family cytokines are also implicated in the control of immune responses to bacterial or fungal infections.

Several infectious diseases have demonstrated the involvement of IL-1 family cytokines. In sepsis, an increased level of IL-1β can contribute to the development of systemic inflammation and organ dysfunction [18,19]. Elevated levels of the IL-1 family of cytokines have been observed in patients with viral infections, such as influenza and HIV, and autoimmune disorders like rheumatoid arthritis (RA) and inflammatory bowel disease (IBD) [20,21].

#### 2.1.2. IL-1 Family and Innate Immune System

The IL-1 family of cytokines plays an important role in the activities of the innate immune system, which is the first line of defense against pathogenic microbes. The innate immune system acts as a rapid response system, providing immediate protection against invading microorganisms before the adaptive immune response is fully activated.

IL-1α and IL-1β are pro-inflammatory and are produced during infection, tissue damage, or other inflammatory stimuli such as exposure to allergens, toxins, or stress. These cytokines act by binding to the IL-1 receptor (IL-1R), initiating a signaling pathway leading to the production of other inflammatory mediators and recruitment of immune cells to the infection or injury site [22,23]. IL-1Ra is a competitive inhibitor of IL-1α and IL-1β, preventing their binding to the IL-1R and dampening the inflammatory response.

IL-18, another IL-1 family member, plays a role in the regulation of both innate and adaptive immunity [24]. IL-18 promotes the production of interferon-gamma (IFN-γ) and activates natural killer (NK) cells and T cells [25,26]. IL-18, IL-1β, and the P2X7 receptor are interconnected in the context of immune signaling. One of the downstream effects of activation of IL-18 and IL-1β is the production of ATP, which acts as a danger signal. ATP released into the extracellular space can then bind to the P2X7 receptor, leading to P2X7 receptor activation [27,28].

**Figure 1:**
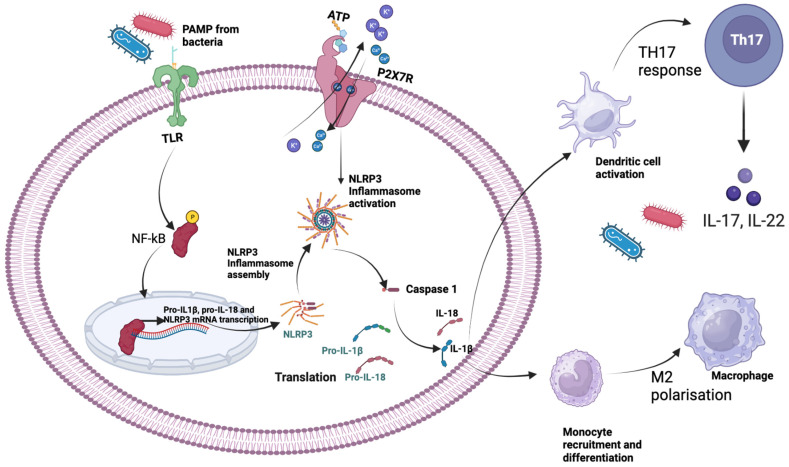
Pro-inflammatory cytokine production and immune consequences. This activation triggers the recruitment of adaptor molecules, such as MyD88 which induces the activation of NF-κB. Nuclear translocation of NF-κB causes the expression of pro-inflammatory genes like pro-IL-1β and NLRP3 mRNA. Simultaneously, the activation of TLRs also triggers the assembly of NLRP3 inflammasome. This multiprotein complex, comprising NLRP3, ASC, and procaspase-1, is responsible for the processing and activation of inflammatory cytokines, like IL-1β and IL-18. Furthermore, the release of ATP from damaged cells or bacteria acts as a danger signal. ATP binds to the P2X7R, leading to its activation. P2X7R activation induces the formation of a pore, allowing the influx of ions, such as calcium, and the efflux of potassium. These events trigger the assembly and activation of the NLRP3 inflammasome, subsequently leading to the maturation and secretion of IL-1β and IL-18. Secretion of these cytokines results in immune cell recruitment and differentiation for a specialized immune response, such as TH17 response secreting IL-17 or IL-22 from dendritic cells or IL4 and IL10 from M2 polarized macrophages.

There is emerging evidence of crosstalk between Toll-like receptors (TLRs) and the P2X7 receptor, highlighting the interconnectedness of these two important components of the innate immune system. TLRs are a group of receptors expressed by different immune cells that identify specific pathogen-associated molecular patterns (PAMPs) derived from microbes. By binding to their respective ligands, TLRs initiate signaling that induces the production of inflammatory cytokines like IL-1β and antimicrobial factors such as phospholipase A2, defensins α and β, and lysozyme [29]. TLR activation by pathogenic PAMPs such as lipopolysaccharides of bacteria causes activation of NF-κB with subsequent transcriptional activation of *NLRP3* and pro-IL-1β mRNAs, which is followed by the NLRP3 protein translation (Figure 1). The NLRP3 inflammasome is a cytosolic protein complex that plays a crucial role in regulating inflammation and immunity in response to cellular stress. Activation of the P2X7 receptor by ATP further causes activation of the NLRP3 inflammasome assembly. The NLRP3 inflammasome then activates caspase 1, the protease that cleaves pro-IL-1β to matured IL-1β [30].

High concentrations of ATP are released during cell damage or inflammation, with implications in immune-mediated responses consequent upon P2X7 receptor activation [31]. IL-1β serves as a multifaceted component in the immune response against HIV infection. IL-1β can be generated and released by immune cells like macrophages and dendritic cells upon contact with HIV antigens, potentially leading to the activation of pyroptosis, an inflammatory form of programmed cell death [15]. This process can release viral particles and inflammatory signals, intensifying the immune response and aiding viral dissemination. Furthermore, IL-1β contributes to inflammation, a pivotal component in the battle against HIV [8]. However, persistent inflammation in HIV can inadvertently activate immune cells susceptible to HIV infection, accelerating disease progression. Additionally, IL-1β can stimulate CD4+ T cells, the primary targets of HIV, influencing viral replication and immune responses [32]. IL-1β contributes to chronic immune activation, a hallmark of HIV infection, which may result in T cell exhaustion and disease progression [33].

On the contrary, IL-1 regulates polyamine metabolism in immune cells modulating immune cell functions and adding to its repertoire of immune regulatory functions. Polyamines, including spermidine and spermine, are pivotal for cell growth, proliferation, and differentiation [34]. In immune cells, IL-1 is found to regulate polyamine metabolism in various ways. It can upregulate enzymes involved in polyamine biosynthesis, boosting polyamine levels crucial for rapid immune cell proliferation during immune responses [35,36]. Furthermore, IL-1’s impact on polyamine metabolism supports tissue repair and wound healing by promoting immune cell proliferation and migration to damaged areas [37]. This multifaceted influence on polyamine metabolism underscores the diverse functions of cytokines like IL-1 in shaping immune responses and cellular processes beyond traditional immune signaling roles.

#### 2.1.3. Diagnostic and Therapeutic Potential

The measurement of members of IL-1 cytokines in clinical settings holds potential for diagnostic and prognostic purposes. Increased levels of IL-18 and its binding protein have been linked to the severity and progression of infectious diseases such as malaria while IL-1β and IL-6 are associated with sepsis and viral infections like COVID-19 [38,39,40]. Monitoring IL-1 family cytokine profiles may aid in identifying patients at risk of developing severe complications and guide therapeutic interventions.

Targeting the IL-1 family of cytokines has emerged as a promising diagnostic and therapeutic approach in infectious diseases. Quantifying the expression patterns of different IL-1 family members in blood or other biological fluids can serve as diagnostic markers. In terms of therapeutics, several drugs have shown efficacy in inhibiting specific IL-1 family cytokines. For example, in the context of infectious diseases, anakinra, and canakinumab have been used successfully to inhibit IL-1β and treat conditions such as sepsis and septic shock [41,42,43]. These drugs bind to IL-1 receptors, preventing the harmful effects of IL-1β. Additionally, the use of IL-1Ra has shown promise in infectious diseases like tuberculosis [44], where it can help modulate the excessive inflammatory response. It is worth noting that ongoing research is dedicated to further exploring the diagnostic and therapeutic potential of targeting the IL-1 family in infectious diseases [45] and consulting with healthcare professionals is crucial for personalized treatment decisions.

As stated earlier, IL-1 is a highly potent proinflammatory mediator that plays a crucial role in immune defense and immune-mediated diseases. IL-1 encompasses two distinct cytokines, IL-1-α and IL-1β, that signal via IL-1R. A related IL-1 family member, IL-1 receptor antagonist (IL-1RA), serves as an inhibitor that competes with both cytokines for binding to the receptor [46].

In infectious diseases, IL-1 is involved in the inflammatory response. Chronic hypoxia and low-grade cell death signals stimulate IL-1 production [47]. IL-1 has been considered a potent soluble cardio-depressant factor during sepsis as it impairs β-adrenergic receptor signaling downstream of the receptor as a consequence of cytoplasmic calcium regulation [46,48]. Importantly, the negative inotropic effects of IL-1 are reversible. Anakinra, an IL-1 receptor antagonist, has been explored as a potential treatment for infectious diseases, including severe sepsis to modulate the inflammatory response and reduce organ damage [46]. By blocking the action of IL-1, these drugs can help to control the inflammatory response that can lead to organ damage in severe infections.

### 2.2. IL-6 Family

The IL-6 family plays a crucial role in immune regulation and inflammation. This family comprises IL-6, IL-11, IL-27, oncostatin M (OSM), leukemia inhibitory factor (LIF), cardiotrophin-1 (CT-1), ciliary neurotrophic factor (CNTF), cardiotrophin-like cytokine (CLC), and neuropoietin (NP). These cytokines have a common gp130 receptor subunit, which is important for signal transduction. IL-6, the prototypical member of the family, is expressed by different cell types, such as immune cells, endothelial cells, and fibroblasts, during infection, tissue damage, or inflammation [49,50,51]. IL-6 is an important mediator of the acute-phase response during infection. It acts by binding to the IL-6 receptor (IL-6R), which is made up of IL-6Rα and gp130 subunits. This complex activates intracellular pathways, such as the Janus kinase–signal transducer and activator of transcription (JAK/STAT) pathway, leading to the production of acute-phase proteins (APPs), immune cell activation, and tissue repair [11,52]. IL-6 causes inflammation through the production of APPs, like C-reactive protein, and activation of immune cells, like T and B cells, and macrophages. It also influences the differentiation and function of T helper (Th) cell subsets, contributing to the regulation of adaptive immune responses during infections [12,52,53].

The dysregulation of IL-6 cytokines has been found in various infectious diseases. Elevated levels of IL-6 have been reported in severe infections, such as sepsis and viral infections, and are associated with poor outcomes [54,55]. Similarly, dysregulated IL-11 signaling is implicated in viral infections, like respiratory syncytial virus (RSV), HIV, dengue virus, and hepatitis C [56,57]. Similarly, IL-6 is involved in the regulation of immune response to viral infections and is associated with the onset of cytokine storm syndrome associated with severe COVID-19 cases [58,59].

#### 2.2.1. IL6 Family in the Innate and Adaptive Immune System

IL-6, which is mainly secreted by neutrophils, plays an important role in innate immune defense against infections. IL-6R activates Notch, regulating its expression in the hemogenic endothelium (HE) and in hematopoietic stem cells (HSCs) [55,60]. This soluble form of IL-6R (sIL-6R) can enter the bloodstream and travel to inflamed areas. Consequently, IL-6 trans-signaling affects cell types such as endothelial cells, epithelial cells, smooth muscle cells, and fibroblasts, leading to the release of different chemokines [61,62]. This, in turn, attracts monocytes and macrophages to promote the resolution of inflammation.

As highlighted earlier, IL-6 is widely recognized for its role in the production and secretion of APPs in response to inflammation. IL-6 has been identified as the primary inducer of the hepatic acute phase response. APPs play diverse and intricate roles in the body’s defense mechanisms, and IL-6-mediated production of multiple mediators in the liver is crucial to combating bacterial pathogens like *Klebsiella pneumoniae* and *Streptococcus pneumoniae* [63,64]. In a *Listeria monocytogenes* infection model, selectively inhibiting IL-6 trans-signaling did not affect APP expression or bacterial load. However, blocking the classic and trans-signaling IL-6 pathways using neutralizing antibodies resulted in reduced APP expression and increased bacterial colony formation in the spleen and kidney [65]. These findings suggest that classic IL-6 signaling plays a primary role in host defense against bacterial infections.

Considering T cell-dependent immune response, CD4+ T helper cells were initially divided into two subsets: TH1 cells, which are responsible for clearing intracellular pathogens, and TH2 cells, which serve as defense against extracellular pathogens and facilitate antibody production by B cells. Later mice studies identified a group of IL-17-producing TH cells, TH17 cells, which undergo clonal expansion facilitated by IL-23, suggesting an additional subset [66,67]. Combined stimulation with TGFβ and IL-6 induced the generation of TH17 cells, while stimulation with TGFβ alone led to the production of regulatory T (Treg) cells [68,69]. IL-6 plays a role in maintaining balance in the differentiation of these two cell types. In humans, differentiation of TH17 cells requires stimulation with TGFβ in the presence of IL-6 and either IL-23 or IL-21. IL-6 does not only affect the TH17 cells and Treg cells balance but also influences the activity of cytotoxic T cells and other T Helper cell subsets. TH17 cells are essential in defending against fungal and bacterial infections, particularly those caused by Candida species [70,71]. Therefore, TH17 cells are an important immune cell subset that should be a focus when studying therapies targeting fungal infections. Thus far, there have been no reports of such infections in clinical trials and post-marketing investigation of tocilizumab, a drug that inhibits IL-6 signaling.

In chronic human viral infections like HIV and hepatitis B virus (HBV) or infections like lymphocytic choriomeningitis virus (LCMV), IL-6 signaling is crucial in controlling the viral activities [55,72]. It stimulates CD4+ T cells, leading to the activation of the germinal center and enhanced antibody production. Interestingly, in addition to IL-6, IL-27 from the gp130 family is also vital for viral control. IL-27 exhibits both interferon-like STAT1-mediated and STAT3-dependent activities that are linked to interferon and gp130 responses, respectively [73,74]. In HBV infection, IL-6 produced by hepatic Kupffer cells inhibits hepatocyte nuclear factor-1α (HNF1α) and HNF4α expression in the liver cells [75,76]. These transcription factors are required to facilitate the gene expression and replication of the HBV. As such, IL-6 plays a protective role in different viral infections. This should be taken into consideration in deciding therapeutic approaches involving IL-6 blockade.

#### 2.2.2. Diagnostic and Therapeutic Potential

The measurement of IL-6 family cytokines like IL-6, in blood or other biological fluids, may have diagnostic potential for identifying patients with viral infections and other inflammatory conditions. High IL-6 levels have been found in COVID-19 patients, which may aid clinicians in the identification of at-risk patients for severe disease [77,78]. Additionally, targeting IL-6 family cytokines may have therapeutic potential in the treatment of inflammatory and infectious diseases. For example, tocilizumab, a monoclonal antibody that targets the IL-6R, has been approved for treating severe COVID-19 and associated cytokine release syndrome, which is also a complication of certain cancer treatments [79,80].

IL-6 is produced by a variety of cell types, including lymphocytes, monocytes, and fibroblasts. In COVID-19, infection by SARS-CoV induces a dose-dependent production of IL-6 from bronchial epithelial cells [81]. This can lead to COVID-19-associated systemic inflammation and hypoxemic respiratory failure, as indicated by elevated blood levels of IL-6, C-reactive protein (CRP), D-dimer, and ferritin [82]. In severe cases of COVID-19, patients often suffer from an overreaction of the immune system, which is caused by the release of other cytokines such as IL-1β and TNF-α in a condition called cytokine storm [83].

IL-6 blocking drugs, tocilizumab and sarilumab, have been trialed to suppress this overreaction [84]. Tocilizumab and sarilumab are anti-IL-6 receptor monoclonal antibodies that have been evaluated in hospitalized patients with COVID-19 who had systemic inflammation. Intravenous tocilizumab has been approved by the Food and Drug Administration Agency (FDA) for the treatment of COVID-19 in hospitalized adults who are receiving systemic corticosteroids and require supplemental oxygen, non-invasive ventilation (NIV), or mechanical ventilation [85].

The World Health Organization (WHO) has also updated its patient care guidelines to include IL-6 receptor blockers for patients who are severely or critically ill with COVID-19, especially when administered alongside corticosteroids [86]. These drugs reduce the odds of death compared with standard care, translating to fewer deaths per thousand patients in critically ill patients [87].

## 3. Anti-Inflammatory Cytokines

Anti-inflammatory interleukins are characterized by their ability to suppress pro-inflammatory signaling pathways and dampen immune responses (Table 2). These interleukins utilize different mechanisms to inhibit inflammation and production of pro-inflammatory cytokines and regulate immune cell activation and function. Some notable anti-inflammatory cytokines discussed here include IL-1Ra, IL-4, and IL-10.

### 3.1. IL-1Ra Roles in Mitigating Inflammation

During infection, IL-1Ra serves as a crucial negative feedback mechanism to limit excessive inflammation and tissue damage. Firstly, as a natural inhibitor of IL-1R, IL-Ra causes inhibition of pro-inflammatory cytokine production by preventing IL-1 binding to IL-1R. This blocks IL-1R downstream signaling, which is required for the production of other inflammatory cytokines such as IL-6. Similarly, IL1Ra prevents excessive cytokine production in response to the mRNA vaccine [6,9]. IL-1 plays a pivotal role in activating immune cells, including neutrophils and macrophages, which are crucial for host defense but can also contribute to tissue damage. IL-1Ra inhibits the activation and recruitment of these immune cells, preventing excessive inflammation and tissue injury [9,95].

One of the ways IL-1Ra exhibits its anti-inflammatory activities is by modulating endothelial cell activation. During infection, endothelial cells lining blood vessels can become activated and promote leukocyte recruitment to the site of infection. IL-1Ra can attenuate endothelial cell activation induced by IL-1, thereby reducing leukocyte adhesion and extravasation, and consequently limiting inflammation [15]. Similarly, IL-1Ra also promotes tissue repair. Inflammation is a necessary process to eliminate pathogens, but excessive or prolonged inflammation can impair tissue repair. IL-1Ra helps to resolve inflammation and facilitate tissue healing by counteracting the inflammatory effects of IL-1, allowing the restoration of normal tissue function [96].

#### Diagnostic and Therapeutic Applications of IL-1Ra

IL-1Ra demonstrates significant diagnostic and therapeutic potential in the realm of infectious diseases. As a diagnostic tool, measuring IL-1Ra levels serves as a biomarker for assessing the severity of inflammation during infections. Elevated IL-1Ra levels in the blood or at the infection site indicate ongoing inflammatory processes. Monitoring changes in IL-1Ra levels can also provide insights into the effectiveness of anti-inflammatory therapies targeting IL-1 signaling pathways.

In terms of therapeutics, IL-1Ra offers valuable anti-inflammatory effects by naturally antagonizing IL-1 signaling. By blocking IL-1 from binding to its receptor, IL-1Ra mitigates excessive inflammation and reduces tissue damage associated with infectious diseases. This mechanism makes IL-1Ra a promising therapeutic option. Currently, there are several IL-1Ra drugs available for clinical use including Anakinra in the treatment of rheumatoid arthritis [96]. IL-1Ra also suppresses the production of inflammatory cytokines, chemokines, and adhesion molecules. This modulation helps restore immune balance and promote the resolution of inflammation during infections. Furthermore, IL-1Ra has the potential to protect against organ damage caused by infections, since there is evidence that it protects tissues against damage from chemotoxicity [91,97]. Its ability to inhibit IL-1-mediated inflammatory processes can help preserve tissue integrity and function.

Combination therapy involving IL-1Ra may be a potential therapy to improve treatment outcomes in infectious diseases. IL-1Ra may be used in conjunction with other therapeutic agents, such as antimicrobial agents or immunomodulatory drugs, to provide synergistic effects in controlling infections and reducing inflammation.

### 3.2. IL-4 as an Anti-Inflammatory Cytokine during Infections

IL-4 is involved in regulating immunity against various pathogens, particularly parasites and bacteria. It facilitates the differentiation of naive T cells into Th2 cells, which secrete IL-4 and other cytokines to enhance humoral immunity and activate eosinophils [98]. Conversely, IL-4 suppresses the differentiation of Th1 cells responsible for cellular immunity and inflammation. In infectious diseases, IL-4 exhibits a dual function: while it can protect against certain infections by stimulating antibody production and eosinophil activity, it can also hinder the elimination of intracellular pathogens by suppressing Th1 responses and macrophage activation [92,99]. IL-4 also suppresses the production of inflammatory proteins like cyclooxygenase-2 (COX-2) and inducible nitric oxide synthase (iNOS) and pro-inflammatory chemokines, like IL-8, CCL2, CCL3, CCL4, and CCL5, which regulate immune cell recruitment to the infection site, thereby controlling immune cell trafficking and reducing excessive inflammation [100,101]. Excessive inflammation can be detrimental to the host, leading to tissue damage and exacerbating the disease. IL-4’s ability to suppress the production of pro-inflammatory chemokines helps maintain a balanced immune response, preventing an overzealous immune reaction that can harm healthy tissues.

IL-4 also promotes B cell class switching to produce IgE antibodies, which not only have a role in allergic reactions but contribute to host defense against parasites and certain infections, facilitating pathogen neutralization and modulation of immune responses [93,102]. When exposed to IL-4, B cells undergo class switching, a process where they change the type of antibody they produce. In the case of IL-4 stimulation, B cells switch to producing IgE antibodies [103]. IgE antibodies are particularly effective against parasites, such as gastrointestinal helminths, by triggering immune responses that lead to the expulsion or destruction of these pathogens. IgE antibodies interact with specific receptors on basophils and mast cells, priming them for immediate immune activation upon encountering the parasite’s antigens. This activation leads to the secretion of potent mediators, such as cytokines, histamine, and enzymes, which promote inflammation and target the parasites for elimination.

#### IL-4 in Disease Diagnosis and Treatment

From a diagnostic perspective, IL-4 can serve as a biomarker for assessing the immune response during infections. Measurement of IL-4 levels in patient samples, such as blood or tissue specimens, can provide valuable insights into the type and intensity of the immune response. Elevated IL-4 levels may indicate an ongoing Th2 immune response, which is typically associated with certain parasitic infections and allergic reactions [104,105]. By monitoring IL-4 levels, clinicians can gain valuable information about the nature and progression of the infection, aiding in diagnosis and treatment decisions.

Therapeutic targeting of IL-4 has shown promise in infectious diseases. Monoclonal antibodies against IL-4 or its receptor have been developed as therapeutic agents. For example, dupilumab targets the IL-4R alpha subunit and has been approved for treating atopic dermatitis and asthma, which are conditions associated with dysregulated IL-4 responses [106,107]. In infectious diseases, blocking IL-4 signaling may have therapeutic benefits by modulating immune responses and reducing inflammation. However, it is crucial to consider the specific pathogen and the type of immune response elicited, as IL-4 can have both protective and detrimental effects depending on the context. High IL-4 levels have a significant impact on the modulation of effector elements that alter the physiology of the intestine, creating an unfavorable environment for worm parasites [108,109]. The administration of IL-4 from an external source can effectively treat persistent worm infections, whereas the use of IL-4 antagonists hinders the protective responses against such infections [110,111].

While the diagnostic and therapeutic potential of IL-4 in infectious diseases is promising, further research and clinical research are required to fully evaluate its role and optimize treatment strategies. The availability of drugs targeting IL-4 and its receptor provides a foundation for exploring its clinical utility. As our understanding of IL-4-mediated immune responses continues to evolve, harnessing the diagnostic and therapeutic potential of IL-4 may lead to more effective management of infectious diseases, ultimately improving patient outcomes.

### 3.3. IL10 and Its Anti-Inflammatory Roles

IL-10 is a potent anti-inflammatory cytokine that plays a crucial role in modulating immune responses during infectious diseases. It acts as a key regulator of immune homeostasis and serves to mitigate excessive inflammation, tissue damage, and immune-mediated pathology [112,113]. The anti-inflammatory roles of IL-10 are important in controlling the immune response and promoting the host’s defense against pathogens.

In the context of infectious diseases, IL-10 helps to prevent immunopathology and limit tissue damage caused by the immune response [90,114,115]. Excessive inflammation can sometimes lead to collateral tissue damage, and IL-10 acts as a safeguard by counterbalancing the inflammatory response. It is particularly important in chronic infections, where prolonged inflammation can have detrimental effects on the host.

IL-10 dysregulation is implicated in different infectious diseases. In some cases, the dysregulation of IL-10 in infectious diseases allows pathogens to escape the host’s immune responses, causing persistent infections [116,117]. Examples include Mycobacterium tuberculosis, which induces IL-10 production to suppress excessive inflammation while hindering immune cell function [118]. Helicobacter pylori exploits IL-10 to suppress the immune response and persist in the stomach [119], and Leishmania parasites induce IL-10 production, impairing immune cell activation and promoting chronic infection [120]. In HIV infection, elevated IL-10 levels contribute to immune suppression, viral persistence, and disease progression [121]. This regulatory response aims to manage the excessive inflammation and immune activation that are characteristic features of HIV pathogenesis. IL-10 is produced by various immune cells, including regulatory T cells (Tregs), monocytes, and macrophages, particularly within the gut mucosa, which is a crucial site of immune activity in HIV infection [122]. Regulatory T cells (Tregs) are known to be a source of IL-10 during HIV infection. These specialized immune cells have immunosuppressive properties and can secrete IL-10 as part of their function. In HIV infection, there is often an expansion of PD-1+ Tregs expressing the programmed death receptor, especially in the gut-associated lymphoid tissue (GALT), where they contribute to the regulation of immune responses [123]. However, excessive IL-10 production by Tregs can also hinder the host’s ability to control the virus effectively. As such, these cells express PD-1, allowing them to interact with PD-L1 on other immune cells, thereby suppressing effector T cell activity [124]. Conversely, in the gut, IL-10 produced in the right amount by Tregs, monocytes, and various immune cells helps immune suppression and inflammation dampening, exemplifying the pleiotropic nature of IL-10 [125]. Understanding how pathogens influence the expression of IL-1 family cytokines is crucial for developing effective diagnostic and therapeutic strategies to prevent adverse effects.

#### IL-10 in the Treatment of Infectious Diseases

IL-10 is crucial for developing strategies to enhance host defense and combat infectious diseases. Targeting IL-10 dysregulation may offer therapeutic potential in restoring immune function and improving disease outcomes. However, harnessing the anti-inflammatory properties of IL-10 also has therapeutic potential. Strategies to enhance IL-10 production or delivery, or to mimic its effects, are being explored as potential interventions in infectious diseases such as *Streptococcus pneumoniae* infection [126]. In addition, the use of IL-10-expressing viral vectors or administration of recombinant IL-10 as a therapeutic approach to control excessive inflammation and limit tissue damage has been explored [127,128].

IL-10 has shown potential in the treatment of infectious diseases due to its ability to regulate immune responses and mitigate excessive inflammation [90,112]. However, using IL-10 as a therapeutic agent is still being investigated, and no specific IL-10-based drugs are currently approved for clinical use in infectious diseases. Nevertheless, several approaches have been explored to harness the immunomodulatory properties of IL-10.

One strategy involves using recombinant IL-10 (rIL-10) as a therapeutic agent. rIL-10 has been studied in various infectious disease models, such as bacterial infections, viral infections, and parasitic infections [89,90,129]. Preclinical studies have demonstrated its ability to suppress pro-inflammatory cytokine production and reduce tissue damage in infection-induced inflammation [90,125]. Similarly, in COVID-19 infection, IL-10 resistance produces adverse effects. IL-10 operates through the induction of a SHIP1-STAT3 complex. This complex moves to the nucleus, leading to the suppression of macrophage activation and the resolution of inflammatory colitis in mice [130]. SHIP1 agonist, with an anti-inflammatory “IL-10 mimetic,” effectively inhibited macrophage activation and resolved colitis in IL-10 receptor knock-out mice. Consistent with these findings, an older study established that small-molecule SHIP1 agonists could overcome IL-10 resistance induced by high glucose in macrophages [131]. As such, it appears that the disruption of normal SHIP1-STAT3 complex formation could be a mechanism contributing to IL-10 resistance. Importantly, SHIP1 agonists seem potentially promising in circumventing this resistance, thereby reducing inflammation. This leads to intriguing speculation that pharmaceutical agents targeting SHIP1 signaling might have a role in alleviating the adverse effects of cytokine storms, presenting a potential therapeutic avenue for COVID-19.

Another approach is to enhance endogenous IL-10 production or signaling. This can be achieved by using agents that stimulate IL-10 production in immune cells or promote IL-10 signaling pathways. For example, certain Toll-like receptor (TLR) agonists, such as TLR2 and TLR4 agonists, have been shown to induce IL-10 production, leading to the dampening of inflammatory responses in infection models [132,133]. Additionally, small molecules such as the salt inducible kinase (SIK) inhibitors that enhance IL-10 signaling by targeting its receptor or downstream signaling molecules are being explored as potential therapeutic options [134].

Although no specific IL-10-based drugs are currently available for the treatment of infectious diseases, various existing drugs indirectly modulate IL-10 levels or activity. For example, glucocorticoids, such as corticosteroids, can induce IL-10 production while suppressing pro-inflammatory cytokines [135,136]. Nonsteroidal anti-inflammatory drugs (NSAIDs) are also shown to upregulate IL-10 expression. Additionally, certain immunomodulatory drugs, such as thalidomide and lenalidomide, have been found to enhance IL-10 production and attenuate inflammatory responses [137,138].

## 4. Interleukins as Biomarkers of Clinical Importance

Interleukins, as biomarkers with clinical utility, hold significant promise in the early and precise diagnosis of various diseases, thus playing a crucial role in improving treatment outcomes. This is especially critical in conditions where early detection remains a formidable challenge, such as infectious disease. One of the primary functions of interleukins as biomarkers is their ability to reflect the state of the immune system. In HIV infection, for instance, the decrease in levels of interleukins like IL-2 and IL-13, alongside the elevation of pro-inflammatory cytokines including IL-1, IL-6, and IL-8, serves as a clear indicator of the immune imbalance associated with the disease [139,140,141]. The reduction in IL-2, an essential cytokine for immune regulation, represents an early immunological dysregulation in HIV [139]. This early detection of immune system alterations through interleukin profiling offers clinicians a valuable tool for identifying HIV infection at its nascent stages. Furthermore, interleukins can provide critical diagnostic thresholds for other infectious diseases. For example, the cut-off level of IL-6 at 4000 pg mL^−1^ has been established for tuberculosis with pleural effusions, enabling a more precise diagnosis of this condition [142]. This cut-off level has been found to have considerable predictive and diagnostic value for tuberculosis with pleural effusions, with a sensitivity of 90.6% and specificity of 76.5%. In neonatal medicine, interleukins like IL-6, IL-8, IL-10, and IL-12 are employed as biomarkers for diagnosing conditions such as sepsis [143]. Their specific cut-off values provide clinicians with essential guidance in making accurate and timely diagnoses in neonates, where early intervention is vital. Interleukins also find utility in the diagnosis and management of other diseases beyond infectious ones. For instance, IL-4 upregulation detected through advanced techniques like three-color flow cytometry serves as a valuable indicator in certain clinical contexts [144].

## 5. Crosstalk between Pro-Inflammatory and Anti-Inflammatory Cytokines

The crosstalk between pro-inflammatory and anti-inflammatory interleukins is a complex and dynamic process that influences immune responses and immune homeostasis. Extensive research has shed light on the intricate interplay between these interleukins, providing insights into their regulatory mechanisms and therapeutic potential.

Pro-inflammatory interleukins, such as IL-1β, IL-6, and TNF-α, are key players in initiating and amplifying immune responses during infection or tissue injury [16]. They promote the activation of immune cells and the release of pro-inflammatory mediators. However, anti-inflammatory interleukins, such as IL-4, -IL-10, and IL-13, are crucial regulators that counterbalance pro-inflammatory signals and facilitate the resolution of inflammation [145].

One aspect of the crosstalk between pro-inflammatory and anti-inflammatory interleukins involves negative feedback loops which prevent harmful responses. Pro-inflammatory interleukins can stimulate the production of anti-inflammatory interleukins to limit excessive inflammation. For instance, high expression of IL-1β and IL-6 are linked to reduced IL-10 production and vice versa as a mechanism to suppress the pro-inflammatory response and prevent tissue damage [146]. As highlighted here, it is known that IL-10 acts as a potent suppressor of pro-inflammatory cytokine production by immune cells, thus attenuating immune response during infection and reducing inflammation.

In addition to negative feedback regulation, anti-inflammatory interleukins can directly inhibit the production and activity of pro-inflammatory cytokines. For example, IL-10 exerts anti-inflammatory effects by suppressing the expression of pro-inflammatory cytokines. Studies have demonstrated that IL-10 inhibits the production of IL-1β and IL-6 in various cell types, including macrophages, monocytes, and T cells [147,148]. IL-4 and IL-13 also exhibit anti-inflammatory functions and can downregulate pro-inflammatory cytokine production, particularly from T cells and macrophages [93,94].

Moreover, the crosstalk between pro-inflammatory and anti-inflammatory interleukins influences the differentiation and function of immune cell subsets. Pro-inflammatory cytokines, including IL-1β, IL-6, and TNF-α, promote the development of pro-inflammatory Th cell subsets, including Th1 and Th17, which drive inflammatory responses [70,71]. In contrast, anti-inflammatory interleukins, such as IL-4 and IL-10, play a critical role in promoting the differentiation of anti-inflammatory Th-cell subsets. IL-4 induces the development of Th2 cells, which are implicated in immune responses against parasitic infections and exhibit anti-inflammatory properties [149,150].

Current research continues to uncover the intricacies of the crosstalk between pro-inflammatory and anti-inflammatory interleukins. It highlights the importance of maintaining a delicate balance between these cytokines for proper immune regulation and prevention of excessive inflammation. Dysregulation of this balance is implicated in various diseases, including autoimmune disorders and chronic inflammatory conditions.

Understanding the crosstalk between these interleukins at a molecular level has paved the way for potential therapeutic interventions. Targeting specific interleukins or their signaling pathways holds promise for modulating immune responses and restoring immune balance in inflammatory diseases. Several studies have explored the use of IL-10-based therapies to control inflammation and ameliorate disease severity. For example, pre-clinical studies investigating the administration of IL-10 or IL-10 receptor agonists have shown promising results in inflammatory bowel disease (IBD) [151], where excessive pro-inflammatory cytokines play a significant role in disease pathogenesis. This is particularly relevant to infectious diseases as IBD comprises diseases like ulcerative colitis and Crohn’s disease. Ulcerative colitis and Crohn’s disease patients unfortunately have a higher risk of common infections, viral infections, and gastrointestinal infections [152]. These approaches aim to enhance the anti-inflammatory actions of IL-10 and restore immune homeostasis.

Furthermore, the development of biological agents targeting pro-inflammatory cytokines has provided effective therapeutic options for various inflammatory conditions. For instance, monoclonal antibodies against TNF-α, such as infliximab and adalimumab, have revolutionized the treatment of diseases like RA, psoriasis, and inflammatory bowel disease [153,154]. These agents directly neutralize TNF-α, alleviating pro-inflammatory signals and dampening immune responses.

Although there has been no research focusing on the potential of combining anti-inflammatory and pro-inflammatory cytokine-targeted therapies to achieve synergistic effects and better disease control, studies showing anti-TNF-α, for instance, result in IL-10 induction in IBD [155]. This may provide insight into the co-administration of IL-10 with anti-TNF-α agents in the treatment of inflammatory infectious diseases like colitis and IBD toward enhanced therapeutic outcomes, with reduced inflammation and improved tissue healing. This suggests that a comprehensive approach targeting both pro-inflammatory and anti-inflammatory pathways may offer greater efficacy in managing inflammatory diseases.

The crosstalk between pro-inflammatory and anti-inflammatory interleukins is a vital mechanism that regulates immune responses and maintains immune balance. Dysregulation of this crosstalk can facilitate the development and progression of different inflammatory diseases. Understanding the intricate interactions between these cytokines has opened avenues for targeted therapies aimed at restoring immune equilibrium. The development of IL-10-based therapies and the success of anti-cytokine biologic agents provide promising strategies for managing infectious and inflammatory diseases. Future research will continue to unravel the complexities of this crosstalk and uncover novel therapeutic approaches for immune-mediated disorders.

## 6. Conclusions

This review has provided valuable insights into the complex roles of pro-inflammatory and anti-inflammatory interleukins (ILs) in infectious diseases. The intricate balance between these cytokines is crucial for orchestrating immune responses against various pathogens, and dysregulation of IL-mediated immune pathways can significantly impact disease outcomes.

We highlight the diverse functions of pro-inflammatory ILs, such as IL-1, IL-6, and IL-8, in promoting inflammation and activating immune cells to combat infectious agents. These ILs play pivotal roles in immune cell recruitment, chemotaxis regulation, and antimicrobial response induction. However, excessive or sustained pro-inflammatory IL signaling can lead to tissue damage and chronic inflammation, emphasizing the need for precise regulation and therapeutic interventions.

Conversely, anti-inflammatory ILs, including IL-10 and IL-4, dampen immune responses and facilitate immune tolerance. These cytokines help limit excessive inflammation, attenuate tissue damage, and promote tissue repair. However, a fine balance must be maintained, as an overemphasis on anti-inflammatory ILs may compromise pathogen clearance and increase susceptibility to recurring infections.

This review also highlights the dynamic interplay between pro-inflammatory and anti-inflammatory ILs, emphasizing the importance of their temporal and spatial regulation in infectious diseases. The modulation of IL-mediated immune responses presents exciting therapeutic opportunities. Targeting specific pro-inflammatory ILs, either through antibody-based therapies or small molecule inhibitors, holds promise in controlling inflammation-associated tissue damage. Similarly, augmenting the expression or activity of anti-inflammatory ILs may alleviate excessive inflammation and promote immune tolerance.

Furthermore, the emerging field of IL-based gene therapy provides novel avenues for manipulating IL expression and signaling in infectious diseases. By modulating IL levels or enhancing the responsiveness of immune cells to ILs, gene therapy approaches offer personalized and targeted interventions with the potential for long-term benefits.

In conclusion, this comprehensive review underscores the complex and multifaceted roles of pro-inflammatory and anti-inflammatory ILs in infectious diseases. A deeper understanding of their mechanisms of action, interplay, and therapeutic potential is essential for the development of innovative strategies to combat infections and promote immune homeostasis. Continued research in this field holds great promise for the advancement of precision medicine approaches in infectious disease management.

## Figures and Tables

**Table 1:** Pro-inflammatory Cytokines in Infectious Diseases.

Cytokine	Cell Source	Function in Infectious Disease	Associated Diseases	Reference
Interleukin-1β	Macrophages, monocytes	Induces fever, activates immune response	Sepsis, septic shock, bacterial and viral infections	[2,7]
Interleukin-6	Macrophages, T cells, fibroblasts	Facilitates immune response, promotes inflammation	Sepsis, pneumonia, viral infections	[11,12]
Interleukin-12	Dendritic cells, macrophages	Enhances cellular immune response	Intracellular infections (e.g., tuberculosis)	[13]

**Table 2:** Anti-inflammatory cytokines, mechanisms, and associated diseases.

Cytokine	Cell Source	Function in Infectious Disease	Associated Diseases	Reference
Interleukin-10	Macrophages, regulatory T cells	Downregulates immune response, suppresses inflammation	HIV infection, sepsis, and chronic infections	[88,89,90]
Interleukin-1 Receptor Antagonist (IL-1Ra)	Various cell types	Limits IL-1-mediated inflammation	Rheumatoid arthritis, sepsis, and infectious diseases	[9,44]
Interleukin-4	T cells, mast cells, and basophils	Modulates immune response, promotes antibody production	Parasitic infections, allergies	[91,92]
Interleukin-13	T cells, mast cells, and basophils	Downregulates inflammation, supports tissue repair	Asthma, helminth infections	[93,94]

## Data Availability

No new data were created or analyzed in this study. Data sharing is not applicable to this article.
